# Erotomania in Alzheimer’s Dementia: A Rare but Clinically Important Association

**DOI:** 10.7759/cureus.100055

**Published:** 2025-12-25

**Authors:** Adekemi Odeyemi, Sadia Shafique, Zuleekhah Balogun, Qasim Mirza

**Affiliations:** 1 Psychiatry, North Staffordshire Combined Healthcare NHS Trust, Stoke-on-Trent, GBR; 2 General Practice, North Staffordshire Combined Healthcare NHS Trust, Stoke-on-Trent, GBR

**Keywords:** alzheimer's disease, delusional disorder, dementia psychosis, erotomania, geriatric psychiatry, neurocognitive disorder, secondary erotomania

## Abstract

Alzheimer’s dementia is a common condition in old age psychiatry, typically presenting with memory impairment, confusion and behavioural disturbance. Erotomania is a rare psychiatric syndrome characterised by the fixed belief that another person, usually someone of higher social or economic status, is in love with the individual. The co-occurrence of erotomania in Alzheimer’s dementia is highly unusual. We present a classical case of secondary erotomania in a patient with Alzheimer’s dementia, highlighting the diagnostic challenges and management complexities associated with this presentation. This case emphasises the need for clinicians to recognise atypical delusional experiences in dementia.

## Introduction

Erotomania, also known as delusion of love, is considered a rare condition, but the exact incidence remains unknown [1]. It is a mental health condition that occurs when a person, the subject, is fixated on the idea that another person, the object, is intensely in love with them. The object is often of a higher social or economic status [2]. The condition earned the name De Clerambault syndrome as it was first described by G.G. De Clerambault in 1885 [2], although its first report is linked to Hippocrates.

Erotomania is classified as primary and secondary. The primary type starts de novo with no background organic or mental health condition [3]. The secondary type, however, which is the presentation in the case under review, is usually preceded by an organic or psychiatric illness.

Dementia, on the other hand, is a group of organic brain disorders manifested as a progressive loss in memory, functioning, and associated with abnormal behavioural phenomena [4]. There are different types of dementia, but the commonest is the Alzheimer’s type. Alzheimer’s dementia accounts for two-thirds of all dementia cases, and not less than 10% of people over 65 years of age suffer from this debilitating condition. Manifestation of the symptomatology may vary with the type. However, most patients still present with cognitive impairment, reduced functioning, memory gaps, and behavioural and emotional components.

A study published by the American Medical Association to evaluate the prevalence of symptoms in dementia showed that 36% of patients suffered apathy, 32% depression and 30% agitation [5]. Other frequently occurring psychiatric presentations amongst inpatients include confusion, sleep disturbances, abnormal perceptions, especially visual hallucinations. Patients may also present with unusual firm and false beliefs known as delusions. This occurs in about 29.1% according to another study [6]. The commonest type of delusion seen in a demented patient is paranoid delusions. This delusion may be elicited as suspicion or outright accusation of loved ones for theft. Delusion of love (erotomania) has not been widely reported in patients suffering from dementia. Only a limited number of cases, fewer than 10, were identified during the preparation of this article. Although delusions are relatively common in Alzheimer’s dementia, erotomania remains an exceptionally rare presentation, with only a small number of cases reported in the literature, making its recognition clinically significant and diagnostically challenging.

Usually, erotomania occurs in young adults, and there are very few case studies about elderly people with erotomania. Schizophrenia remains the most frequently reported disease associated with this delusion [2].

## Case presentation

An 80-year-old man with progressive cognitive decline presented with a fixed erotomanic delusion involving a celebrity, associated with functional deterioration, disorientation, and risky behaviours, including attempted travel to meet the perceived love object. Cognitive testing and neuroimaging supported Alzheimer’s dementia, and symptoms improved partially with antipsychotic treatment alongside safeguarding interventions.

Our patient, to be referred to as "Mr X," is an 80-year-old Caucasian man who is a jovial and active member of the community. He retired as a nursing officer and is independent with activities of daily living. He was previously able to drive until three months prior to presentation, when he had three car accidents within a short space of time, approximately six months. Mr X was brought to the hospital emergency unit in September 2022 by the ambulance service following concerns raised by neighbours. His neighbours were worried about his increasing level of confusion. He had become distressed with his gradual but obvious decline in functioning and confided in one of his neighbours about suicidal intent. This prompted escalation to services. He was described as disorientated, unsure of what he was doing and wandering late at night, appearing to be lost. On one occasion, he was seen at the train station trying to get a ticket to travel to the USA to see his "girlfriend," who is a famous American music artist that he believed strongly he was in love with. He had not had any real contact with this lady. She neither calls him nor sends him text messages. The only contact he had with her was merely as a fan on social media. He listens to her music and watches her social media videos, including live streaming.

Mr X had been having challenges with his memory prior to these events. His general practitioner had clearly recorded his memory problems as far back as 2019. During the COVID-19 pandemic, a progressive decline in his memory was reported, including feeding his elderly sister mouldy food. It was also reported that he had not been looking after himself. Concerns that he might have been financially exploited due to his vulnerability were raised by his neighbours as well.

Assessment and investigations

On his arrival at the emergency unit of the hospital, he was examined. He had a series of investigations, including routine bloods such as a full blood count, electrolyte level, thyroid, kidney and liver function test, a urine dipstick and chest X-ray. There were no significant results (Table 1).

**Table 1:** Summary of laboratory investigations at presentation LUC: large unstained cells; HbA1c: glycated haemoglobin; CRP: C-reactive protein; ALT: alanine aminotransferase; GFR: glomerular filtration rate; TSH: thyroid-stimulating hormone; T4: thyroxine

Test	Result	Units	Reference Range	Interpretation
Haemoglobin	141	g/L	120-170	Normal
White Cell Count	6.1	×10^9^/L	4.0-11.0	Normal
Platelet Count	161	×10^9^/L	150-450	Normal
Red Cell Count	4.62	×10^12^/L	4.30-5.70	Normal
Haematocrit	0.437	L/L	0.37-0.51	Normal
Mean Cell Volume	94.5	fL	80-100	Normal
Mean Cell Haemoglobin	30.5	pg	26.5-31.5	Normal
Neutrophils	3.4	×10^9^/L	2.0-7.5	Normal
Lymphocytes	1.8	×10^9^/L	1.10-3.60	Normal
Monocytes	0.6	×10^9^/L	0.20-0.80	Normal
Eosinophils	0.1	×10^9^/L	0.04-0.40	Normal
Basophils	0	×10^9^/L	0.00-0.10	Normal
LUC	0.2	×10^9^/L	0.00-0.44	Normal
HbA1c	47	mmol/mol	20-41	Mildly Elevated
CRP (Wide Range)	<4.0	mg/L	0-5	Normal
Ferritin	140	ng/mL	20-250	Normal
Serum Folate	8.4	ng/mL	3-12	Normal
Serum B12	317	pg/mL	200-900	Normal
Calcium	2.31	mmol/L	2.20-2.60	Normal
Adjusted Calcium	2.51	mmol/L	2.20-2.60	Normal
Inorganic Phosphate	1.3	mmol/L	0.80-1.50	Normal
Magnesium	0.87	mmol/L	0.70-1.00	Normal
Albumin	35	g/L	35-50	Low-Normal
Alkaline Phosphatase	68	U/L	30-130	Normal
ALT	11	IU/L	0-48	Normal
Total Bilirubin	10	µmol/L	0-21	Normal
Sodium	142	mmol/L	133-146	Normal
Potassium	4.9	mmol/L	3.5-5.3	Normal
Urea	6.2	mmol/L	2.5-7.8	Normal
Creatinine	98	µmol/L	59-104	Normal
Estimated GFR	62	mL/min/1.73m^2^	-	Borderline but Acceptable
TSH	0.97	mU/L	0.1-5.0	Normal
Free T4	15	pmol/L	12-23	Normal
Urine Dipstick	Normal	-	-	No Blood, Protein, Ketones, Glucose

**Figure 1:**
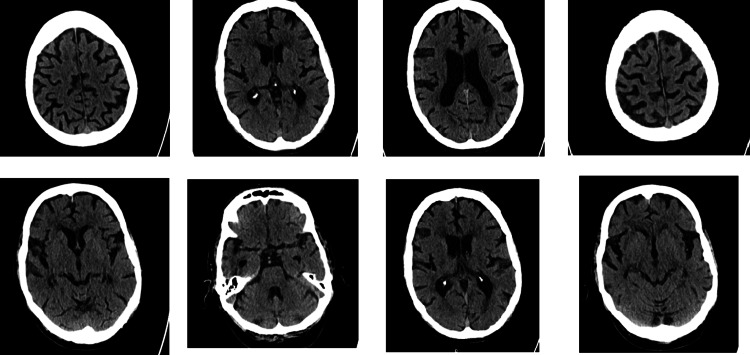
Axial CT head images demonstrating generalized involutional changes and small-vessel disease Axial non-contrast CT head images showing generalised involutional changes, including widened sulci and ventricles, consistent with global cerebral atrophy. Scattered hypodensities in the periventricular and deep white matter are in keeping with chronic small-vessel ischemic disease. No acute intracranial pathology was identified.

A CT head scan was also done, which showed generalised involutional changes with small vessel disease (Figure 1). No acute intracranial pathology was identified.

He was then referred to the Mental Health Liaison Team (MHLT). Following assessment by MHLT, Mr X was recommended for admission under section 2 of the Mental Health Act. Subsequently, he was admitted to the old age unit of the psychiatric hospital.

While on the ward, he was observed to be glued to his phone, constantly watching TikTok videos of the American singer, waving and blowing her kisses. He would sometimes hold the phone close to his ears and talk like he was having a conversation with her. He was so preoccupied with thoughts that this lady was in love with him and that they were dating. Attempts at shaking this belief were abortive. In his opinion, he was not famous or rich and could not exactly pinpoint why the young, famous lady had handpicked him as her suitor. However, he was sure that he was her choice. He went further to state that she requested to marry him, and he said yes. As he remained fixated on this belief, he was actively planning a trip to meet his professed lover in the USA. He was buying things online and searching for a flight ticket. In fact, he ordered a pair of earrings costing £40 to present to her as a Christmas gift. He also demanded to go shopping for a guitar as he intends to join a live band with her in America for a musical concert.

There were no records to show that he had any previous contact with mental health services. Although his general practitioner had suspected a memory problem, there had been no indication to refer to the mental health services until this recent turn of events. Significant past medical history includes epilepsy, hypothyroidism, type 2 diabetes mellitus and hyperlipidaemia. He has a family history of epilepsy, but no family history of mental health problems was recorded. Mr X had been married twice, with both relationships ending in divorce, with no children. His initial medications consisted of dapagliflozin, gliclazide, sodium valproate, levothyroxine, simvastatin, cholecalciferol, Braltus, Ventolin and Fostair inhalers.

Management

At his initial review at the emergency unit of the hospital, a working diagnosis of acute confusional state was made. A behaviour chart detailing hallucinations/delusions was advised.

**Figure 2:**
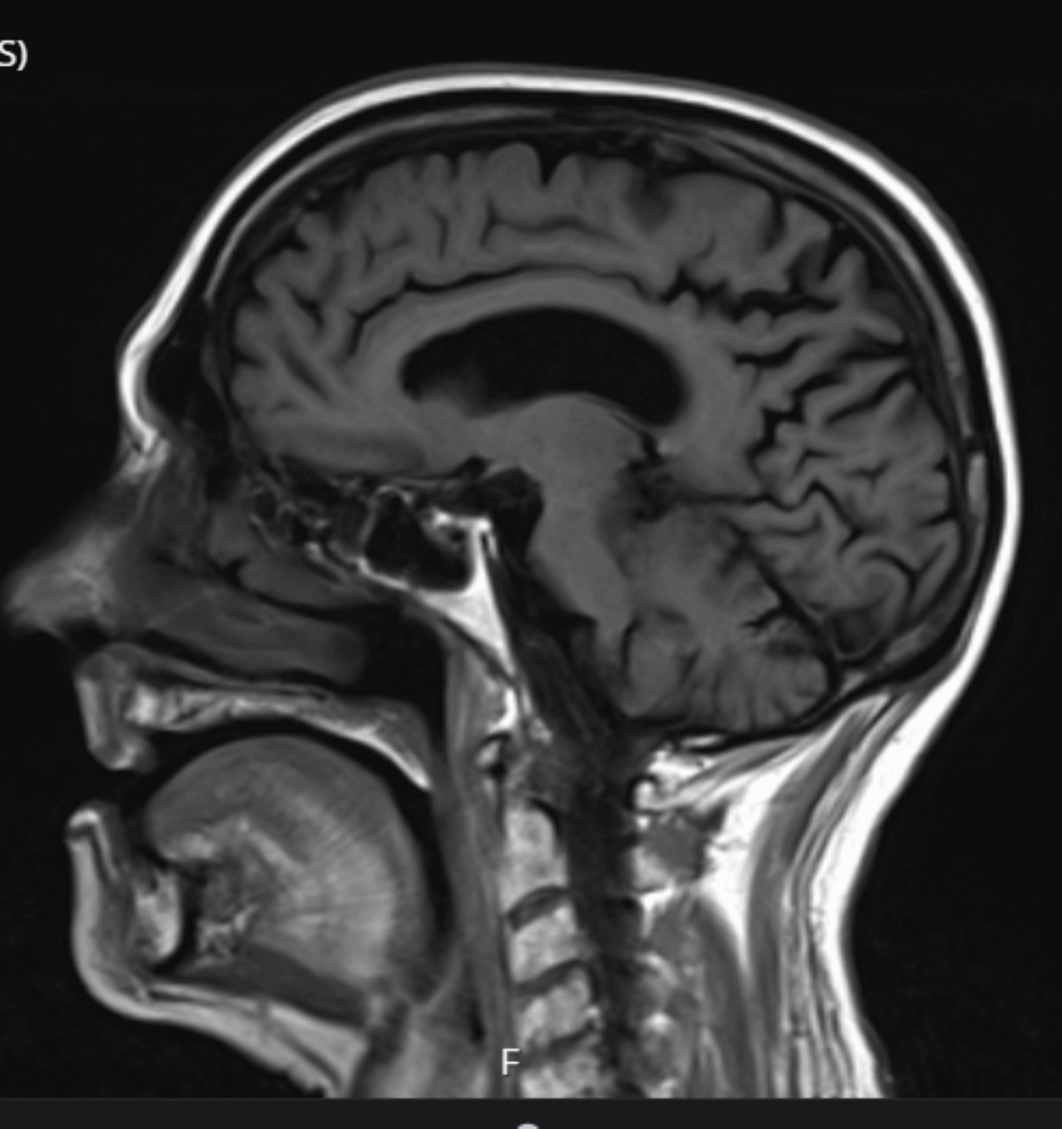
Sagittal T1-weighted MRI demonstrating global age-related involutional changes Sagittal T1-weighted MRI demonstrating cortical thinning and mild ventricular prominence consistent with global involutional changes.

**Figure 3:**
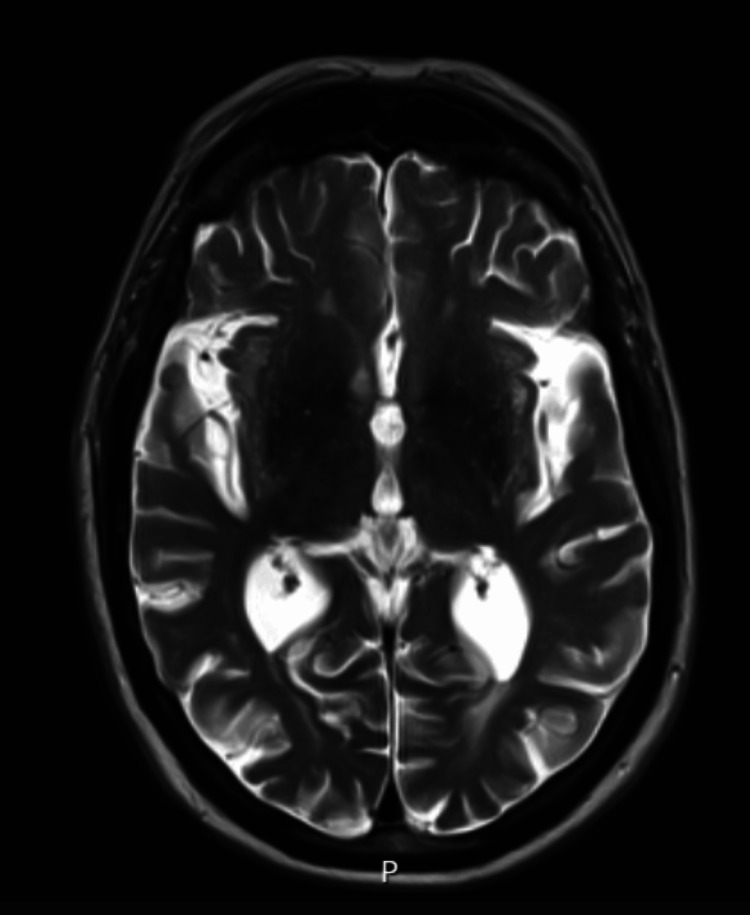
Axial T2-weighted MRI corresponding to reported cortical, ventricular, and hippocampal changes Axial T2-weighted MRI from the patient’s scan, demonstrating widened cortical sulci and ventricular prominence, with hippocampal region volume loss as reported in the radiological assessment.

He was observed to have tremors; hence, a neurology referral was made to rule out Parkinsonism or Lewy body dementia. An MRI scan was advised. This showed global age-related involutional changes. There was moderate bilateral hippocampal atrophy and moderate small vessel disease (Figures 2-3). The MRI findings, particularly bilateral hippocampal atrophy with small vessel disease, supported an Alzheimer’s dementia diagnosis and helped narrow the differential away from frontotemporal dementia. This diagnostic clarification guided management towards treating the erotomanic delusion as secondary to dementia.

While under the care of liaison psychiatry, an impression of mania with psychosis was made. His sodium valproate was increased to 700mg BD, and he was given lorazepam 0.5mg BD. On admission to the psychiatric ward, routine blood tests were done again. He had an ECG done and reviewed. There were no concerns.

An Addenbrooke cognitive examination III (ACE-III) was completed. This showed a total of 62/100 with the following breakdown: attention: 10/18, memory: 14/26, fluency: 5/14, language: 18/26 and visuospatial: 15/16. Overall, the ACE-III profile showed a prominent memory deficit with reduced attention and fluency, supporting an underlying neurocognitive disorder rather than a primary mood disorder. In the context of functional decline and collateral history, this strengthened the working diagnosis of Alzheimer’s dementia and informed the need for a longer-term care and safeguarding plan.

An occupational therapy assessment was requested. This revealed reports of exploitation by some neighbours, prank calling, recording him while assaulting and encouraging him to apply for loans and buy bitcoin. Safeguarding concern was raised, and a social worker referral was made to look into his finances, secure his credit card and bring possible scammers to book.

Mr X was planned to be started on aripiprazole, but due to nationwide shortages at that time, risperidone 0.5mg OD was commenced and later increased to 1mg. At this point, his delusions remained unshakeable, causing a further increase in risperidone to 1.5mg. The prolactin level was measured and found to be elevated at 630 (reference range: 63-262). This informed the decision to taper risperidone down and commence olanzapine. With olanzapine use, a slight improvement was noticeable as he became less preoccupied with his phone; however, his blood sugar shot up. After that, olanzapine was discontinued, and aripiprazole was commenced.

His delusional thoughts started to shake about a week after starting aripiprazole. This was in January 2023. He stated that his acclaimed girlfriend was being nasty as she was involved with a soldier abroad. This stemmed from a duet video of the singer and another artist dressed in an army uniform. To him, this event meant that she was no longer in love with him.

His aripiprazole was gradually increased to 10mg. He began thinking thoughts how to forget about the famous artist and may not want to travel to America. Due to his vulnerability and potential for exploitation, a residential care referral was put in place for him.

## Discussion

Erotomania, already established as an unusual psychotic disorder, is exemplified by an individual's belief that another person is infatuated with them. The object of the delusion is usually beyond reach, being of much higher social or financial status, already married or disinterested [3].

This disorder was categorised as Persistent Delusional Disorder F.22 in the International Classification of Diseases and Related Health Problems, 10th revision (ICD-10) [7]. Evidence from case reports and small clinical series also highlights the rarity of erotomania in dementia populations [3,8]. Additionally, therapeutic outcomes reported in psychogeriatric literature support the effectiveness of atypical antipsychotics in managing such presentations [9].

According to a case series reported by Sowmya et al., the diagnostic criteria for primary erotomania were described using the following: (a) A delusion of being in love with another person. (b) This person is of much higher social status. (c) The other person has initially fallen in love. (d) The other person had made the initial advances. (e) The onset is swift. (f) The love object is constant. (g) The subject explains the unpredictable behaviour of the loved one. (h) The course is chronic. (j) Hallucinations are absent [3]. These criteria can be dubbed for secondary erotomania except that an underlying pathology is identified, and the love object may be shifting.

Our patient fulfils the criteria above. He was clearly deluded about having a relationship with a world-renowned music artist. The object was of a higher societal status, obviously being a celebrity and also more affluent. It is important to note that the subject believed that the popular musician was the one who professed her love to him. The presentation was swift. His object of love was constant. There were no hallucinations. Although initial misconceptions emanated from his constant interaction with his phone, that he was responding to unseen stimuli, this was later understood that he was assuming to be on a call with his love object. He continued to tirelessly explain how his object was in love with him and was awaiting his arrival in America.

An array of differential diagnoses considered included acute confusional state, mania with psychosis, Alzheimer’s dementia, frontotemporal dementia and the delusional syndrome - erotomania. These differentials were gradually tailored down. Frontotemporal dementia was a possibility due to the seemingly sexually disinhibited features, but there was no further clinical or radiological evidence to support this. The sexual theme was also limited to his firm belief in the false relationship.

The diagnosis of a mood disorder remained strongly under review; however, his mood could not be described as persistently elated, irritable or depressed. In addition, he did not fulfil the criteria for diagnosing a mood disorder [3]. His delusions were also not fleeting and lasted for more than six months, only mellowed by medication. He could not be described as grandiose, as he denied feeling important or having any special abilities. He neither appeared energetic nor was he seen to be overtly active. He could not be described as depressed. Most of the time, he was buried in his phone, believing that he was conversing with his star girlfriend. His symptomatology presents as a well-circumscribed belief in this false romantic affair. A diagnosis of Alzheimer’s dementia, mixed type, was established from the clinical assessments and investigations. The poor ACE III score and particularly low performance in the memory subset buttress this. Furthermore, the CT and MRI results were consistent with the diagnosis of Alzheimer’s dementia. Particularly, bilateral hippocampal atrophy is pathognomonic for Alzheimer’s dementia [5,6]. A second diagnosis of erotomania was also made.

## Conclusions

Although the literature on erotomania co-occurring with dementia consists mostly of case reports and small samples of patients, the relationship between dementia and erotomania remains uncertain. Nonetheless, this co-occurrence is not overtly shocking to see, given that misinterpretation of events is common in organic brain disorders.

Antipsychotics, as seen in this case, remains the mainstay of treatment for most delusional disorders. In recent years, there have been reports of positive therapeutic outcomes with atypical antipsychotics. However, emphases should be placed on the need for a biopsychosocial approach to sustain insight. Patients with this presentation would require significant social care input and psychological interventions to explore unresolved conflicts as well as provide necessary reassurance.
